# Pharmacist prescriber smoking cessation intervention during the COVID-19 pandemic

**DOI:** 10.18332/tid/170580

**Published:** 2023-10-27

**Authors:** Trudy Thomas, Bronte Sykes, Shilpa Shah, Sarah Corlett

**Affiliations:** 1 Medway School of Pharmacy, Universities of Greenwich and Kent at Medway, Kent, United Kingdom; 2 Kent LPC, Kent, United Kingdom; 3 Community Pharmacy North East London, London, United Kingdom; 4 Pharmacy Department, Medway NHS Foundation Trust, Gillingham, Kent, United Kingdom

**Keywords:** smoking cessation, smoking, SARS-CoV-2, COVID-19, pharmacy

## Abstract

**INTRODUCTION:**

During the pandemic, smokers who wished to access support to quit faced additional barriers. A smoking cessation service which utilized pharmacist independent prescribers working within community pharmacy was implemented. Clients received behavioral advice via a consultation with an advisor and then three consultations with a pharmacist, who prescribed varenicline, where appropriate. Consultations were by phone or video. This evaluation assessed the self-reported outcomes and experiences of clients and pharmacists.

**METHODS:**

A mixed-methods approach was used involving both on-line questionnaires to clients and interviews with a sample of questionnaire respondents and participating prescribing pharmacists.

**RESULTS:**

The questionnaire was completed by 85 clients with 59% reporting they had quit. Eleven clients and seven out of eight pharmacists were interviewed. Varenicline had been received by 96% of clients. The best aspects of the service reported by clients in the questionnaire and at interview were support received from the pharmacist and ease of access to varenicline. Clients regarded the service as being ‘safe’ to access during the pandemic. Nearly three-quarters of client respondents (72%) stated no service improvements were required. However, national supply challenges made collection of varenicline from the nominated pharmacy an issue. Some clients experienced a long wait-time before accessing the service. For pharmacists, the service offered flexibility including the opportunity to contact clients ‘out of office’ hours without distractions. However, not being physically in the pharmacy could result in them not being able to access the client’s medicine history. Pharmacists identified that remote consultations were not ideal for all clients.

**CONCLUSIONS:**

Pharmacist prescribers can deliver effective smoking cessation services through remote consultations. Greater flexibility would allow the service to be tailored to the client’s need.

## INTRODUCTION

In the early phase of the COVID-19 pandemic, cigarette smokers were recognized to be particularly vulnerable to SARS-CoV-2 infection (COVID-19), as the habit of smoking encourages greater hand-to-mouth contact increasing the risk of transmission from the environment^1^. The enzyme which facilitates the transport of the virus into the cells (angiotensin – converting enzyme 2) is known to be predominant in the lungs of smokers resulting in increased viral load for any given exposure^2,3^. This increased threat was communicated to the UK public via the media^4^, and in July 2020 the Department of Health and Social Care funded the ‘Today is the Day’ campaign which encouraged smokers to quit to ‘protect themselves from increased risk of severe illness from COVID-19’^5^. This may have motivated some smokers to quit or to seek help^6^. However, increased pressure on general practice and social distancing requirements effectively meant that GPs closed their doors^7^ and there were reduced opportunities for smokers to access effective intervention in the form of combined behavioral and pharmacotherapy support with either varenicline, bupropion or nicotine replacement therapy^8,9^.

UK stop-smoking services are commissioned by local health authorities and demonstrate considerable variability between localities in their design and organisation^10^. In Kent, prior to the pandemic, smokers wishing to quit were referred to a specialist service to receive behavioral support from a smoking advisor. Those requiring pharmacological support [varenicline (Champix^TM^)] then were referred for a face-to-face consultation with a pharmacist (non-prescribing) with Champix^TM^ supply via patient group direction (PGD). PGDs enable the legal supply of prescription-only medicines without a prescription. The Champix^TM^ PGD requires the pharmacist to interview patients in person^11^. Whilst pharmacies stayed open during the pandemic, there were limits on the number of people permitted to enter the pharmacy at any one time and initially there were problems accessing personal protective equipment. Thus, during COVID-19 it was not feasible to continue the existing service as it had been delivered. Pharmacist prescribers can prescribe based upon their independent assessment of a patient. Critically, they can review patients’ in-person or using on-line or telephone consultations. An innovative service was therefore designed to enable clients to access smoking cessation pharmacological support, following referral by an advisor, using pharmacist prescribers.

This study aims to evaluate the accessibility, feasibility and acceptability of a remote pharmacist prescriber smoking cessation intervention.

## METHODS

### Study setting and design

This cross-sectional mixed-methods evaluation was conducted between April and July 2021 in Kent, England. It explored client and pharmacists’ views on a new service which utilized pharmacist independent prescribers to supply varenicline to clients of the smoking cessation service through remote consultations.

### Intervention

Within this service, the client received behavioral advice via a remote consultation (phone or video) with a smoking advisor and then had three remote consultations with a prescribing pharmacist over 12 weeks^12^. If varenicline was appropriate, the prescribing pharmacist arranged for supply to be collected from the client’s community pharmacy of choice. This service was delivered for 10 months from June 2020, facilitating access to smoking cessation pharmacological support throughout the most restrictive phases of the English pandemic response. The study period of the evaluation covers the full time the remote service was operational.

### Participants

Invitations to participate in a telephone or on-line interview were distributed to all pharmacist prescribers commissioned by the Local Pharmaceutical Committee (LPC) to provide the service; two follow-up requests were sent to non-responders. Pharmacists were also asked to act as gate-keepers to all clients who had received smoking cessation support between 1 June 2020 and 21 March 2021 by distributing a mobile phone text, including a link to an on-line questionnaire. The request was sent twice. The final section of the questionnaire invited clients to participate in a follow-up interview.

One hundred clients consented to participate in the survey but 15 did not indicate their smoking behavior at the beginning of the program. These individuals were excluded from the analysis. Approximately half of all respondents who completed the questionnaire (40/85; 47%) agreed to participate in a follow-up interview by phone. In order to provide as broad a perspective on the service as possible, from the 40 volunteers, 11 were selected based upon their age, gender, smoking cessation outcome, whether they pay for their prescription, and who referred them into the scheme. Seven of the eight pharmacists who delivered the service agreed to be interviewed. The 8th pharmacist declined due to a lack of time.

### Assessment tools

*Client questionnaire*

This was developed from a survey used in previous evaluations by the research team^13^, adapted to cover all specifications included in the original local smoking cessation service. The resulting questionnaire was reviewed in conjunction with the LPC who had commissioned the remote service. The questionnaire comprised 32 questions distributed across three sections. Section 1 (questions 1–17) covered sociodemographic details and smoking history. Questions were principally of binary response (yes/no) or categorical, e.g. number of cigarettes smoked per day before and after the intervention. Section 2 (questions 18–29) consisted of primarily Likert-style questions requiring the respondent to say how strongly they agreed/disagreed with certain statements addressing views on different aspects of the service, such as convenience of service, satisfaction with the consultation and the information provided on varenicline. Section 3 (questions 30–32) allowed the respondent to give an overall evaluation of the service (excellent to poor) and to include free-type responses on what they liked best about the service and how it could be improved.

*Client and pharmacist interviews*

Semi-structured interviews were carried out to further explore the successes and areas for improvement of the service over Microsoft Teams, Zoom, WhatsApp or the telephone, according to the participant’s preference. They were digitally recorded and transcribed verbatim. Where platforms like Microsoft Teams were employed, the transcript automatically created by the program was used as a basis for the final transcript. Automatically generated transcripts were checked and corrected by a researcher (BS).

Client interview gathered information on clients’ smoking history, whether the service helped them to quit, and their experiences of using this service. The pharmacist interviews aimed to gather information on professional background, usual scope of practice as a prescriber and previous involvement in smoking cessation services. Their views and experience of providing this service were explored.

### Data analysis

IBM SPSS Statistics version 28.0 (IBM Corp, Armonk, NY) was used to perform statistical analyses. A Wilcoxon two-tailed signed-rank test was undertaken to compare number of cigarettes smoked by clients prior to and following the service. Statistical significance was assumed for p<0.05. The data were checked to determine whether they met the assumptions required prior to running the test. Descriptive statistics were used in the assessment of client views of various aspects of the service and the support received from the prescribing pharmacist. Free-text responses to open-ended questions were categorized and coded to establish the proportion of respondent comments related to each category. NVivo 11 was used to support thematic analysis of the interview data and identification of patterns in interviewees’ responses. An inductive approach was taken with themes identified at a semantic level, with a framework approach adopted; such that themes were directed by and reflected the explicit content of the data^14^. All qualitative analysis was performed initially by one member of the research team (BS), and then a 10–20% sample reviewed by another (SC).

The confidentiality and anonymity of participants was assured.

## RESULTS

### Participants

Demographic characteristics for the 85 clients who consented to take part, completed the questionnaire, including their smoking behavior, and of the 11 who undertook interviews, are reported in Table 1. Compared to all questionnaire respondents, fewer interviewees self-referred to the smoking cessation service and a higher proportion self-reported being successful in their quit attempt. However, in terms of their gender, age, and the number of cigarettes smoked per day they were broadly representative. Demographic details have not been provided for the pharmacists, given their limited number, to maintain anonymity.

**Table 1:** Summary demographic characteristics of clients who had received remote smoking cessation support from a pharmacist independent prescriber between 1 June 2020 and 21 March 2021

*Characteristics*	*Survey respondents (N=85)*	*Interviewees (N=11)*
	*n (%)*	*n (%)*
**Gender**		
Female	39 (46)	5 (45)
Male	46 (54)	6 (55)
**Age** (years), mean ± SD	53.0 ± 12.5	49.1 ± 13.7
**Ethnicity**		
White	80 (94)	11 (100)
Asian British/Asian	3 (3.5)	0 (0)
Other	2 (2.5)	0 (0)
**Paid for prescription**		
Yes	34 (40)	5 (45)
No	50 (60)	6 (55)
**Cigarettes per day at start of service**		
≥30	26 (31)	2 (18)
20–29	21 (25)	5 (45)
10–19	24 (28)	3 (27)
<10	7 (8)	1 (9)
Not specified	7 (8)	0 (0)
**Number of previous quit attempts**		
0	5 (6)	0 (0)
1–2	44 (52)	6 (55)
3–5	27 (32)	2 (18)
6–10	8 (9)	2 (18)
>10	1 (1)	1 (9)
**Varenicline prescribed**		
Yes	82 (96)	11 (100)
No	3 (4)	0 (0)
**Successful quit attempt**		
Yes	50 (59)	9 (82)
No	35 (41)	2 (18)
**Referral**		
Self	61 (72)	6 (55)
GP	14 (17)	2 (18)
Other HCP (e.g. midwife, pharmacist)	8 (9)	3 (27)
Other (NHS stop smoking line)	2 (2)	0 (0)
**Consultation**		
Telephone	80 (94)	10 (91)
MS ‘Teams’	1 (1)	1 (9)
Face to face in Pharmacy	4 (5)	0 (0)

The majority of respondents (n=61; 72%) were self-referrals to the smoking cessation service and had previously attempted to quit smoking (n=80; 94%). Only 5 people had not made at least one previous quit attempt. The modal number of failed attempts was two although there was considerable variability around this with one client reporting 12 previous failed attempts. Almost all of the consultations (n=80; 94%) were carried out by telephone^12^.

### Service outcomes

Varenicline was not received by three clients. Reasons for this included that varenicline was out of stock (1), they were pregnant (1), or were taking other medication (1). The majority (60/82; 73%) of those who took varenicline completed the 12-week course. Those that stopped before the 12 weeks did so because it was out of stock (n=2), felt they no longer needed it (n=6), had experienced side-effects (n=4) including sleep problems, anxiety, low mood or suicidal thoughts, one had difficulty getting to the pharmacy to collect the medication, and another had difficulty getting the prescription. Recommencing smoking was reported by six clients as the reason for not continuing the course. Whilst 85 participants reported the number of cigarettes smoked before the service, only 78 reported their smoking behavior after the service, of these, 58 (74%) reported smoking fewer cigarettes following the service with no-one indicating smoking more. The number of cigarettes smoked was significantly lower after than before the service (p<0.001). Quitting smoking was reported by 59% (n=50) of clients by the end of the 12-week program.

### Client views and experiences of the service

Client views from the questionnaire are summarized in Table 2.

**Table 2:** Reported experiences from the questionnaire of clients who received the pharmacist independent prescriber smoking cessation service, 1 June 2020 – 21 March 2021

*Statement*	*Clients n*	*Strongly agree or agree %*	*Neither agree nor disagree %*	*Strongly disagree or disagree %*
I felt comfortable with the pharmacist asking me about the medicines I take	76	95	4	1
The pharmacist helped me to understand how the varenicline could help me	73	92	7	1
The pharmacist helped me to deal with any concerns I had about taking varenicline	73	85	14	1
The pharmacist helped me to understand how the varenicline would affect other medicines that I take	72	83	14	3
I understood everything that was discussed	76	97	3	0
I had an opportunity to ask all the questions I wanted to	76	88	11	1
The pharmacist answered all my questions	72	92	6	2
I would have liked to have more time with the pharmacist	76	14	50	36
I didn’t feel comfortable discussing my lifestyle with the pharmacist	76	21	18	61
It was convenient for the consultation to be carried out remotely	76	89	7	4
It was inconvenient to collect the varenicline from the pharmacy I selected	72	29	15	56
I would recommend this service to other people who wish to give up smoking	72	92	5	3

The overwhelming majority were positive about this service agreeing that they were comfortable talking to the pharmacist about their medicines (95%; n=72) and that the pharmacist helped them to understand how varenicline could support their quit attempt (92%; n=67)^12^. Clients concerns about taking varenicline or how varenicline would interact with other medicines were addressed by the pharmacist (83%; n=60). They agreed that they had sufficient opportunity to ask questions (88%; n=67), any questions they had were answered (92%; n=66), and that the time allocated for the consultation was sufficient (86%; n=65). Respondents were also comfortable talking about their lifestyle (79%; n=60) with the pharmacist. The majority of clients would recommend the service to others (92%; n=66). The remote nature of the consultation was convenient for almost 90% of respondents. The most common negative issue related to the collection of varenicline from the client’s chosen pharmacy with almost one in three (29%) identifying this aspect of the service as being inconvenient.

In section 3, three out of four clients (76%; 56/74) rated the service as excellent overall. Only one respondent stated that they would not recommend the service to a friend.

In response to the free-text questions, clients stated that the best thing about the service was the level of support received and the ease and convenience of the service to access varenicline. Broadening the selection of pharmacies from which the varenicline could be collected, providing an option to collect the varenicline from their GP practice, and delivering the prescription directly to their home address, were suggested as possible improvements.

The themes identified from the client interviews were consistent with the findings from the survey (Table 3).

**Table 3:** Sample quotations from interviews of clients who received the remote pharmacist independent prescribing smoking cessation service between 1 June 2020 and 21 March 2021

*Issues*	*Client quotations*
**Relationships**	
Client with pharmacist	‘He [the pharmacist] was phenomenal. Any questions I had or anything he was just brilliant … I would have to say it [the best thing] was the professionalism and warmth that I got from the pharmacist that actually prescribed the Champix^TM^.’ (Male, aged 27 years, 10–19 cigarettes/day)
Client with pharmacist and smoking support advisor	‘The pharmacy would always check I was OK, and the nurse would also do it. So, I had two people keeping an eye on me, or keeping an ear on me, and it did help having the two.’ (Female, aged 68 years, <10 cigarettes/day)
Client with pharmacist	‘[The pharmacist] gave me all their details and said if I had any issues just to call them straight away.’ (Male, aged 53 years, 20–29 cigarettes/day)
**Access to varenicline**	
Medicine availability	‘Yeah, they basically said there was a nationwide shortage so we can’t get any. I’m not kidding, … No other information or nothing.’ (Male, aged 53 years, 20–29 cigarettes/day)
Delays in access	‘If you are someone that’s smoking, let’s say, 60 a day and your health is not in a good place and you really want to give up, I think having a three or four months’ lead time between applying and hearing back, anything could happened in that time. And by that time, the person might go “Well you know, I wanted to give up but now I’m back in the full flow of it, so don’t worry about it”’. (Male, aged 27 years, 10–19 cigarettes/day)
Ease of collection	‘Every time I went to pick up my next kind of batch of Champix^TM^, you would think that I was trying to purchase an illegal substance. It was ridiculous how hard it was to pick up.’ (Male, aged 27 years, 10–19 cigarettes/day)
**Remote consultations**	
Convenience - client	‘Like I said, if you’re working full-time … having an appointment with you GP is a bit of a nightmare. But being able to do this service over the phone was a lot easier for me because I could be at work and take that call. It was never a problem at all.’ (Male, aged 50 years, 20–29 cigarettes/day)
Safety concerns/ client - infection	‘I didn’t miss them at all [face-to-face consultations] and I think that given the pandemic, to be honest with you, it actually made me feel safer.’ (Female, aged 56 years, 20–29 cigarettes/day)
No objective reassurance of effect	‘So that was the only difference for me this time, not being able to see my nicotine levels coming right down to zero … I missed having that ...’ (Female, aged 47 years, ≥30 cigarettes/day)

### Support to quit

*Relationships*

Nine of the eleven interviewees explicitly expressed that they were impressed or grateful for the support received. Having both the pharmacist and the smoking advisor to encourage them to keep on track was deemed helpful and beneficial. The words ‘friendly’ and ‘approachable’ were used countless times to describe the service providers^12^. Some had shared their work mobile number so that if the client was facing any issues they could reach out for help.

*Access to varenicline*

Many respondents believed having access to varenicline was the reason they were able to quit smoking. However, three clients were unable to complete their course of treatment as the pharmacies were unable to fulfil their prescription due to a nationwide shortage of Champix^TM^. This supply issue, whilst outside of the pharmacist’s control, did leave some clients disappointed. Clients reflected that communication specifically about this issue could have been improved. Furthermore, some clients had to wait a considerable time to access the service following their initial enquiry, potentially affecting their motivation to quit.

### Ease and convenience of service

*Remote consultation*

Clients when interviewed also reported that the best things about the service were the convenience and ease of remote consultations. Several alluded to how much easier this aspect of the service was compared to their expectations. They appreciated that the remote nature of the consultation enabled them to arrange their appointments within their work schedule. It was easier for clients to commit to and perceived as being safer during the pandemic.

Some clients did suggest that having an option for face-to-face consultations may be beneficial, dependent upon the individual’s circumstances and personal preference. Physical tests, such as measuring nicotine levels or lung capacity, were mentioned as being useful motivational tools. However, clients recognized that this would not have been possible at the time of the pandemic; the thought of going to a GP surgery for a face-to-face consultation during the pandemic was quite anxiety-inducing for some, and this may have prevented them from seeking help.

A minority of clients found it inconvenient to collect the varenicline from their selected pharmacy either because it was a long way from home or because parking near to the pharmacy was difficult. Suggestions for how to improve the service therefore included broadening the choice of pharmacies from which the prescription could be collected. Three clients also had had negative experiences when they had collected their medicine. One client described an over-zealous procedure for checking identity when collecting the medicines, which reduced the motivation to continue with the service.

### Pharmacist views and experiences

The pharmacists’ views on the service broadly reflected those of their clients (Table 4).

**Table 4:** Sample quotations from interviews with pharmacists who delivered the remote pharmacist independent prescribing smoking cessation service, 1 June 2020 – 21 March 2021

*Issues*	*Pharmacist quotations*
**Relationships**	
Pharmacist with client	‘To develop a relationship with the patient so that when you’re calling them, they’re able to tell you exactly how they’re feeling, exactly their side-effects, exactly what’s going on … even sometimes you get them ask questions about drugs that are not Champix^TM^, but because they have access to a pharmacist and they can ask those questions as well.’ (Pharmacist 5)
Pharmacist with client	‘They can ask you what worries them, they can ask you if they’re having interrupted sleeping patterns. You know, whereas before they can ask the advisor, but the advisor cannot give them an in-depth explanation of how it works, how they can tweak it, to make it work for them, so they can still have a good sleep at night.’ (Pharmacist 5)
Pharmacist with advisor	‘I get quite a few advisors asking me for my advice to when they have certain patients. And this is something that hadn’t happened before … and certainly now we’re on first name basis, you know, they phone me for information, or we’re emailing each other, and rather than saying here’s a client, you know it’s kind of a conversation now … So it’s built a different type of network and it’s a different net worth for the patient, because now we’re working in collaboration rather than isolation delivering the service.’ (Pharmacist 3)
**Remote consultations**	
Convenience – pharmacist	‘You know when I was doing face-to-face, we would give someone a time and say look turn up at two o’clock and I’ll see you. But I don’t know what two o’clock looks like, unpredictability from my end, so at two o’clock am I getting 10 people come through my door? … And then the worst-case scenario I find the appointment time come, but I’m so busy that I’m not wholly focused on it.’ (Pharmacist 2)
Flexibility – both pharmacist and client	‘When people missed appointments, whether they had, you know they were overrun on work or whatever it was, when they missed appointments that’s when they went back on the smoking. With remote consultation, that’s not, you know, it’s not an option because we were flexible. And if they were overrun, they would just ring the next day or I’d ring them in the evening and we were on track.’ (Pharmacist 3)
Safety concerns – pharmacist – access to medical records whilst off-site	‘The biggest hindrance was not being able to access summary care records off premises. So, you know, a lot of patients knew the tablet colors but didn’t know what the tablets were for. […] so being able to access summary care records remotely out of the premises would be a huge advantage to take things further in this service and other services.’ (Pharmacist 3)
Not suitable for everyone	‘When we are able to offer face-to-face service that should be offered for some people – I’m thinking people with hearing difficulty, mental health, those sort of people need face-to-face.’ (Pharmacist 7)
**Future service developments**	
	‘Now when I’m ringing patients, they’re not picking up straight away whereas before when they were in lockdown, there was a different issue, so that flexibility, not flexibility but kind of more of a controlled environment may be needed now.’ (Pharmacist 3)

*Relationships*

The relationship between the pharmacist and client was thought to be key to the success of the service. The pharmacists perceived their deeper knowledge and understanding of how varenicline works empowered them to recommend small changes to the treatment plan, such as managing issues related to sleep patterns, which the smoking advisor would not have been able to do.

A positive change in the dynamic between the advisor and the pharmacist was achieved as a direct consequence of the new service with a perceived shift towards a more collaborative relationship, where the two professionals were working together for the client.

*Ease and convenience of service*

Pharmacists considered that the convenience of the service for clients was key to its success. The remote nature was thought to be particularly useful for mothers and younger people with busy social and working lives, as the flexibility of the remote service enabled clients to be contacted outside normal working hours. This was beneficial for the pharmacists too, as the unpredictable nature of community pharmacy makes scheduling appointments within normal working hours challenging. Contacting clients out of hours, for example in the evening and on Sundays, meant that the pharmacist could complete the consultation without interruption.

Being fully remote was also thought to be beneficial for more nervous or anxious clients who may have felt uncomfortable at a face-to-face meeting, but were able to engage more openly with the pharmacist at a distance.

*Remote consultation*

The pharmacists also emphasized that due to the nature and flexibility of the remote service those who missed appointments could simply have the consultation re-scheduled. Pharmacists believed that this flexibility contributed to the overall success rate. However, as the UK eased out of lockdown and ‘normal’ life resumed, pharmacists did report experiencing greater difficulties with clients missing appointments and being difficult to reach. Therefore, whilst the flexibility of the service was beneficial, pharmacists suggested that a stricter schedule would minimize wasting time that they could better spend on other services.

The major concern regarding the service related to access to the client’s medical records (summary care records) which was not possible if the pharmacist contacted the client out of hours when they were not physically in the pharmacy. If clients were not able to give an accurate medical history this then raised concerns about the safety of prescribing varenicline. Pharmacists believed that having access to summary care records both on and off site would allow them to address any issues and make appropriate decisions in a timely manner. Whilst the remote nature of the service was very convenient, the pharmacists recognized that it was not always optimal for all clients and that there should be an option of face-to-face appointments.

## DISCUSSION

The findings of this study suggest that a pharmacist prescriber remote smoking cessation service based around supply of varenicline is a feasible and acceptable service for people wanting to quit, with its accessibility offering benefits to service users and providers. This fulfilled a need at the time of the pandemic, but offers a possible option for smokers post-COVID-19.

The service was very well received with both the support received by the healthcare provider and prompt access to varenicline through the prescribing pharmacist being major facilitators to the success of clients’ quit attempt. The quit rate of 59%, self-reported without any objective measurement, and of variable duration following the end of the 12-week program, was slightly higher than the normally observed quit rate of 20–40%^6^. However, these findings concur with reports, also using self-reported quit rates, that smokers in the UK were more likely to attempt to quit after the first lockdown (April 2020) compared to before^6^. Despite offering the service by telephone or through video consultations, the majority selected the telephone. These findings again reflect the national picture; smokers were more likely to engage with remote smoking cessation support from any provider, with the telephone being the preferred mode^6^. NHS digital have linked the preference to patient unfamiliarity with video conferencing technology and convenience^15^. Clients and pharmacists particularly liked the flexibility and convenience of the remote consultations. However, pharmacists recognized that a hybrid approach, whereby they could see some people in person and others remotely, would mean that the service could be adapted to the particular needs of the individual. For example, a consultation with a client with hearing difficulties or communication problems, such as a stroke survivor, may be more effective face-to-face^16^. This potentially opens up smoking cessation services for a wider range of the population.

Varenicline reduces cravings and increases quit success^17,18^. During the study period (June 2020–March 2021) supply issues with varenicline resulted in a few clients not completing the 12-week program. In June 2021, Pfizer, the manufacturer of varenicline, halted its distribution and a class 2 recall was subsequently announced in October 2021 by the medicine regulator^19^. Varenicline remains unavailable and current national prescribing guidance has been updated in January 2023 to recommend that nicotine replacement therapy is offered to provide pharmacological support, where required. It also recommends that community pharmacists are involved in local smoking cessation campaigns^9^.

The contribution of community pharmacists to support the delivery of healthcare services in response to COVID-19 has been recognised^7^. Pharmacies comparative accessibility to the public often meant that they were the first port of call for people seeking advice about their health but unable to make an appointment with their GP, dentist or optician. Pharmacists managed an increased demand for prescription medicines with over-the-counter consultations and other extended roles, such as administering vaccinations, with some of these services not being formally contracted by the Government.

The pharmacists who delivered the smoking cessation service valued its flexibility as it enabled them to contact clients outside of normal working hours, meaning they could carry out their consultations without being interrupted. Interruptions and distractions within the pharmacy are recognized as a contributing factor in dispensing-errors and ‘near-misses’^20^. Therefore, completing consultations without interruptions is desirable. However, pharmacists whilst off premises could not access the client’s medical record (summary care record). This could increase the likelihood of a prescribing error occurring if the client was unable to provide an accurate medical history^19^.

From 2026, graduates who have completed a pharmacy degree in the UK will register with the regulatory body as independent prescribers. Work needs to be done to ensure that there will be integrated care pathways to maximize use of these highly qualified individuals to support the overburdened NHS^21^. NHS England is supporting qualification of existing pharmacists as prescribers, including those working in community pharmacies to facilitate service delivery. IT solutions need to be explored to allow prescribing pharmacists to have read/write access to the patient’s summary care record to permit documentation of pharmacy conducted interventions.

### Strengths and limitations

Respondents were not asked whether they had COVID-19 which could have influenced their motivation to quit. However, a study conducted in the UK during the first 12 months of the pandemic confirmed that no difference was observed in quit attempts and in the motivation to quit, between those who had or had not contracted the infection^22^. Furthermore, whilst the number of people who took part in the study was small, clients from all over Kent participated and over half of those who completed the questionnaire volunteered for interview; a purposive sample of clients were invited for interview reflecting the broad demographic characteristics of the questionnaire sample. Those interviewed included the one participant who completed the questionnaire who stated that he/she would not recommend the service to friends. Seven of the eight pharmacists who provided this service were also interviewed. Interviews were undertaken by a psychology graduate (BS) to enable independent and unbiased evaluation of the service provided by the pharmacists. The impact of varenicline on the success of this service cannot be separated from the other components. It is not possible to know if a remote pharmacist prescribing service based around nicotine replacement therapy would have shown similar results and further study is needed in this area.

### Implications

Prior to COVID-19, a remote service which facilitated access to pharmacological support for smoking cessation would not have been feasible or acceptable to the general public. Post COVID-19, many more healthcare services in the UK and world-wide offer remote consultations under the banner of ‘telehealth’ and for many interactions including lifestyle interventions outcomes are as good as face-to-face consultations^23,24^. Remote consultations offer convenience for clients and providers alike and potentially save journeys offering a cost effective and climate conscious way to support people. However, it is acknowledged that meeting someone in person often provides more information than can be gathered over the phone. By capturing facial expressions and body language, useful non-verbal communication tools, the true emotions and feeling of clients can be determined. Equally, for those with hearing difficulties or mental health problems, face-to-face appointments may be preferred. Therefore, these consultations should not replace face-to-face consultations completely but could act as an adjunct service providing accessible, tailored and expert support to those motivated to quit.

## CONCLUSIONS

Utilizing pharmacist prescribers working within community pharmacy to provide remote consultations with smokers interested in quitting during the COVID-19 pandemic enabled pharmacological support with varenicline, the adjunct of choice at the time of the study, to be provided. The service was well received by clients. NHS England should explore contracting pharmacist prescribers to provide similar services in the future. Without further action and investment, particularly in primary care, the UK Government will fall short on its ambition of being smoke-free by 2030^25,26^.

## Data Availability

The data supporting this research are available from the authors on reasonable request.
